# The Role of GLU_K5_-Containing Kainate Receptors in Entorhinal Cortex Gamma Frequency Oscillations

**DOI:** 10.1155/2008/401645

**Published:** 2008-11-17

**Authors:** Heather L. Stanger, Rebekah Alford, David E. Jane, Mark O. Cunningham

**Affiliations:** ^1^Institute of Neuroscience, Faculty of Medical Sciences, Newcastle University, The Medical School Framlington Place, Newcastle upon Tyne NE2 4HH, UK; ^2^The Molecular Biology, Biochemistry and Bioinformatics (MB3) Program, Towson University, Room 360, Smith Hall, 8000 York Road, Towson, MD 21251-0001, USA; ^3^Department of Physiology and Pharmacology, MRC Centre for Synaptic Plasticity, School of Medical Sciences, University of Bristol, University Walk, Bristol BS8 1TD, UK

## Abstract

Using in vitro brain slices of hippocampus and cortex, neuronal oscillations in the frequency range of 30–80 Hz (gamma frequency oscillations) can be induced by a number of pharmacological manipulations. The most routinely used is the bath application of the broad-spectrum glutamate receptor agonist, kainic acid. In the hippocampus, work using transgenic kainate receptor knockout mice have revealed information about the specific subunit composition of the kainate receptor implicated in the generation and maintenance of the gamma frequency oscillation. However, there is a paucity of such detail regarding gamma frequency oscillation in the cortex. Using specific pharmacological agonists and antagonists for the kainate receptor, we have set out to examine the contribution of kainate receptor subtypes to gamma frequency oscillation in the entorhinal cortex. The findings presented demonstrate that in contrast to the hippocampus, kainate receptors containing the GLU_K5_ subunit are critically important for the generation and maintenance of gamma frequency oscillation in the entorhinal cortex. Future work will concentrate on determining the exact nature of the cellular expression of kainate receptors in the entorhinal cortex.

## 1. INTRODUCTION

KARs are made up of various combinations of five subunits: GLU_K5_, GLU_K6_, GLU_K7_, KA1, and KA2 [1, 2] which are abundantly expressed in the neocortex [3]. These subunits make up tetramers of either homomeric or heteromeric assemblies, with GLU_K5–7_ being able to form functional homomeric receptors [1, 4]. KA1 and KA2 cannot form functional receptors when expressed alone [5, 6], yet are able to form functional KARs when expressed heteromerically with other subunits [1, 7, 8]. Differential patterns of expression of KARs in the CNS coupled with the existence of splice variants and mRNA editing suggest complex neurophysiological roles for the various subunits, and different roles in neuronal networks depending on their localization [9, 10].

Of particular interest is the role of KARs in the generation and maintenance of network neuronal oscillatory activity in cortical regions [9, 10]. Gamma frequency oscillations occur between 30–80 Hz and have been observed in many areas of the brain, including the hippocampus [11–13] and cortical regions [14–16]. Cortical gamma oscillations are important in higher brain functions, such as learning, memory, and cognition [17–19], as well as processing of sensorimotor information [15, 16, 20]. In carrying out these functions, cortical gamma oscillations are implicated in various central processes, including long-term potentiation (LTP) and synaptic plasticity [21], with important roles in temporal regulation of neuronal activity.

Gamma frequency oscillations are recordable from the MEC during wakefulness in humans [22, 23], as well as in vivo in rodents [12], in vitro from perfused guinea pig brains [24, 25], and isolated rat brain slices [26, 27]. These gamma oscillations in the MEC play a role in the formation, processing, storage, and retrieval of memories [17, 18]. Previously it has been demonstrated in an in vitro preparation of the MEC that application of nanomolar concentrations of kainate (200–400 nM) can induce persistent gamma frequency oscillations [26–28]. Using this in vitro model of MEC gamma frequency oscillations it has been elucidated that this activity is primarily generated by inhibitory-based neuronal networks [29–31]. A similar mechanism for the generation of gamma frequency activity has been demonstrated in both the hippocampus and neocortex [32, 33].

To date our understanding of the role of the KARs in neuronal network activity has been hampered by a paucity of selective pharmacological agents. The competitive AMPA/KAR antagonist, 2,3-dihydroxy-6-nitro-7-sulfamoyl-benzo[*f*]quinoxaline (NBQX), shows little selectivity between AMPA receptors and KARs at high concentrations, yet at low concentrations (1 *μ*M) can be used to block AMPA receptors, and isolate KAR responses [34, 35]. However, NBQX shows no selectivity between different KAR subunits. The role of GLU_K5_ and GLU_K6_ subunits in neuronal oscillatory activity in the hippocampus has been previously investigated using receptor knockout mice [9, 10]. However, interpretation of work using transgenic models should be viewed in the light of the knowledge that compensatory factors may play a role. The recent development of pharmacological agents with specificity for distinct subunits has led to the possibility of a detailed pharmacological investigation of the role of specific KARs in cortical gamma frequency oscillations. (*S*)-3-(2-Carboxybenzyl)willardiine (UBP302) is a novel selective GLU_K5_-containing KAR antagonist, with activity at both homomeric and heteromeric GLU_K5_-containing receptors [36, 37]. The activity of UBP302 on GLU_K7_ is controversial, Dolman et al. [37] showed that UBP296 (racemic form of UBP302) only weakly inhibited [^3^H]kainate binding to human GLU_K7_ (K_*i*_ value of 374 ± 122 *μ*M). However, in an electrophysiological assay UBP302 was found to block rat homomeric GLU_K7_ receptors with an IC_50_ value of 4 *μ*M but at a concentration of 100 *μ*M only very weakly blocked rat GLU_K6_/GLU_K7_ receptors [38]. 5-Carboxy-2,4-di-benzamido-benzoic acid (NS3763) is another novel glutamate antagonist, which is selective and noncompetitive for homomeric GLU_K5_-containing KARs [39, 40]. (*RS*)-2-amino-3-(3-hydroxy-5-tert-butyl-isoxazol-4-yl)propanoic acid (ATPA) is a selective GLU_K5_-containing receptor agonist [41]. ATPA has been shown to depress excitatory and GABAergic synaptic transmission in the hippocampus [42, 43]. However, Cossart et al. [35] demonstrated that lower concentrations of ATPA could directly depolarise hippocampal GABAergic interneurons leading to increases in the levels of tonic inhibition onto pyramidal neurons. More recently, similar concentrations of ATPA to that used in the Cossart et al. [35] study have been shown to facilitate both evoked and action potential-independent glutamate release in the neocortex [44].

The data presented here demonstrates a role of GLU_K5_-containing KARs in the MEC by examining the contribution of these receptor subtypes to gamma frequency oscillations. Using a pharmacological approach, we have demonstrated that GLU_K5_-containing KARs are important for the maintenance of gamma frequency oscillations in the MEC. Moreover, the selective activation of GLU_K5_-containing KARs can induce persistent gamma frequency oscillations in the MEC. We also demonstrate that it is the specific activation of homomeric GLU_K5_-containing KARs that is important for the generation of gamma frequency oscillations in the MEC.

## 2. MATERIAL AND METHODS

### 2.1. Preparation of EC-hippocampal slices

All procedures involving animals were carried out in accordance with UK Home Office Legislation. Male Wistar rats, weighing >150 grammes, were first anaesthetised by inhalation of the volatile anaesthetic isofluorane. This was immediately followed by intramuscular injection of a terminal dose of ≥100 mg/kg ketamine and ≥10 mg/kg xylazine. After confirmation of deep anaesthesia, rats were intracardiacally perfused with ∼50 mL sucrose-modified artificial cerebral spinal fluid (aCSF), composed of (in millimolar (mM)): 252 sucrose, 3 KCl, 1.25 NaH_2_PO_4_, 2 MgSO_4_, 2 CaCl_2_ *·*2H_2_O, 10 glucose, and 24 NaHCO_3_. All salts were obtained from BDH Laboratory Supplies (Poole, UK), except MgSO_4_ which was obtained from Sigma Chemical Co (Mo, USA).

The whole brain was rapidly removed and maintained in a bath of cold sucrose-modified aCSF (4-5°C) during the dissection procedure. Horizontal slices (450 *μ*m thick) were cut using a vibroslice (Leica VT1000S). Transverse EC-hippocampal slices were then transferred either to a holding chamber or directly to the recording chamber. They were maintained at 32 ± 1°C, at the interface between a continuous perfusion (~2-3 mL/min) of NaCl-based aCSF (containing (in mM): 126 NaCl, 3 KCl, 1.25 NaH_2_PO_4_, 1 MgSO_4_, 1.2 CaCl_2_ *·*2H_2_O, 10 glucose, 24 NaHCO_3_) and humidified carbogen gas (95% O_2_/5% CO_2_). Slices were allowed to equilibrate for 60 minutes before any recordings were taken.

### 2.2. Electrophysiological recording and drug application

Extracellular recordings were taken using glass electrodes pulled from borosilicate glass capillaries (GC129 TF-10, 1.2 mm OD/0.94 mm ID) (Harvard Apparatus, Kent, UK) using a Flaming/Brown micropipette puller, model P-97 (Sutter Instrument Co., Calif, USA). This created electrodes with resistances of 2–4 MΩ. Electrodes were filled with NaCl-based aCSF and positioned in Layer III of the MEC. Control readings were taken from slices before drug application to confirm that any network activity seen following treatment was due to the presence of drugs.

To evoke gamma frequency oscillations, 400 nM kainic acid ((2*S*,3*S*,4*S*)-3-carboxymethyl-4-(prop-1-en-2-yl)pyrrolidine-2-carboxylic acid; Tocris Cookson, Bristol, UK) was bath applied to EC-hippocampal slices and left to equilibrate for 2-3 hours or until gamma oscillations had stabilised. All other drugs were bath applied to slices at known concentrations: UBP302 ((*S*)-3-(2-carboxybenzyl)willardiine; gift from Dr. David Jane, Department of Pharmacology, University of Bristol, UK) at 10 *μ*M; ATPA ((*RS*)-2-amino-3-(3-hydroxy-5-*tert*-butylisoxazol-4-yl)propanoic acid; Tocris Cookson, Bristol, UK) at 1–5 *μ*M; NS3763 (5-carboxy-2,4-di-benzamido-benzoic acid; Tocris, Bristol, UK) at 10–15 *μ*M; NBQX (2,3-dihydroxy-6-nitro-7-sulfamoyl-benzo[*f*]quinoxaline; Tocris, Bristol, UK) at 1–10 *μ*M; and Carbachol (Sigma, UK) at 10–20 *μ*M.

### 2.3. Data acquisition

An AppleMac computer with the Axograph OSX software package (AxographX, Dr. John Clements, Australia) was used for all data acquisition. Signals were analogue filtered at 0.01–0.3 kHz and then digitized at a frequency of 10 kHz. Power spectra were constructed, where power at a given gamma frequency was defined as the area under the peak between 20 and 80 Hz. Power spectra were generated from digitized data, using 60 second epochs of recorded activity, and it was from these spectra that values for gamma oscillation peak frequency, peak amplitude, and spectral area power in the gamma frequency band were obtained.

### 2.4. Data analysis

Data analysis was carried out using Excel and Kaleidagraph software packages. Kaleidagraph software was used to generate pooled power spectra, and the Excel package was used to calculate the mean and standard error of mean (SEM) of results, and to draw up histograms and line graphs. All data is presented as mean ± SEM. SigmaStat (Systat software, USA) was used for all statistical tests. Normality tests were carried out, and if data was found to be normally distributed, two-tailed paired *t-tests* were run. However, if data failed the normality test, the Wilcoxon signed rank test was carried out. This provided us with *P*-values for all data sets, and the significance level was set at 95%; values less than *P* = .05 were deemed to be statistically significant.

## 3. RESULTS

### 3.1. Induction of kainate-driven gamma oscillations in the MEC

**Figure 1:**
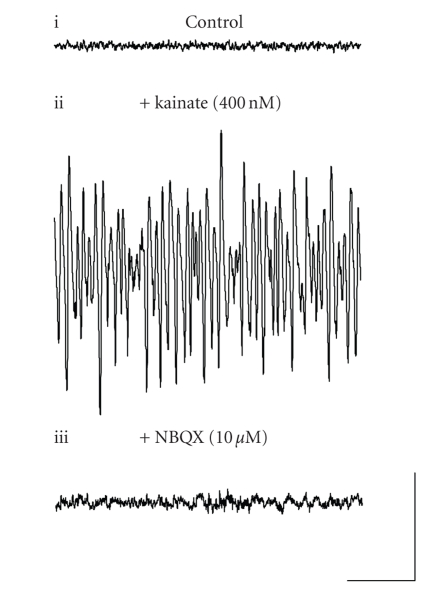
Gamma frequency oscillations can be induced in layer III of the MEC by application of kainate. (a) Extracellular field recordings showing 1 second epochs of activity (i) in control setting, (ii) following application of 400 nM kainate, and (iii) following application of 10 *μ*M NBQX in the presence of 400 nM kainate. Scale bar represents 200 milliseconds and 100 *μ*V.

Previously, it has been demonstrated that low concentrations of kainic acid (kainate) evoke gamma frequency activity in the rat MEC in vitro [26, 27]. In this investigation, we produced persistent gamma oscillations in the MEC by bath application of kainate (400 nM) (Figure 1). Robust gamma frequency oscillations (39.4 ± 1.6 Hz; *n* = 17) were evoked in layer III of the MEC in all slices to which kainate had been applied (*n* = 17). This activity was generated within 15 minutes of kainate superfusion, a stable baseline was observed after 60–90 minutes. As previously reported [19], application of the competitive AMPA/KAR antagonist NBQX (10 *μ*M) effectively abolished these kainate-induced gamma oscillations (*n* = 3) (Figure 1).

### 3.2. A role for GLU_K5_-containing KARs in the maintenance of kainate-driven gamma oscillations

**Figure 2:**
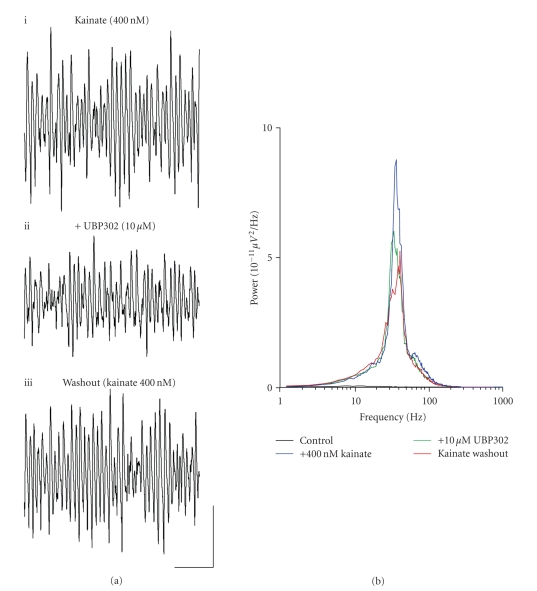
Antagonizing GLU_K5_-containing KARs with UBP302 inhibits kainate-driven gamma frequency oscillations in the MEC. (a) Extracellular field recordings showing 1 second epochs of activity (i) in the presence of 400 nM kainate, (ii) following 10 *μ*M UBP302 application, and (iii) during a washout period into 400 nM kainate. (b) Pooled power spectra (*n* = 9) produced from 60 second epochs of extracellular field recorded data, showing a control recording (black), a recording in the presence of 400 nM kainate (blue), application of 10 *μ*M UBP302 (green), and washout back into 400 nM kainate (red). Scale bar represents 200 milliseconds and 100 *μ*V.

A possible role for GLU_K5_-containing KARs in the maintenance of kainate-driven gamma frequency oscillations in the MEC was investigated by testing the ability of the GLU_K5_ selective antagonist, UBP302, to inhibit preestablished kainate-induced gamma activity. Gamma oscillations were generated in the MEC by bath application of kainate (400 nM) and allowed to stabilise (*n* = 9) (Figure 2(a)). In the presence of UBP302 (10 *μ*M), the amplitude of kainate-induced gamma oscillations was significantly reduced (control, 116.7 ± 44.1 *μ*V^2^/Hz; *v.* UBP302, 70.0 ± 30.3 *μ*V^2^/Hz; *P* < .05; *n* = 9), and area power of oscillations was also significantly decreased (control, 1586.0 ± 503.3 *μ*V^2^/Hz.Hz; *v.* UBP302, 1155.1 ± 441.4 *μ*V^2^/Hz.Hz; *P* < .05; *n* = 9) (Figures 2(a), 2(b)). However, following UBP302 application, the frequency of oscillations remained unchanged (control, 40.4 ± 2.1 Hz; *v.* UBP302, 38.9 ± 2.6 Hz; *P* > .1; *n* = 9). Washout of the effects of UBP302 on gamma frequency oscillations could not be achieved (*n* = 9) (Figures 2(a), 2(b)).

### 3.3. A role for GLU_K5_-containing KARs in the generation of gamma oscillations in the MEC

To investigate the role that GLU_K5_-containing KARs may play in the induction of kainate-driven gamma oscillations, we carried out two experiments, using the selective GLU_K5_-containing KAR agonist, ATPA, and antagonist, UBP302.

**Figure 3:**
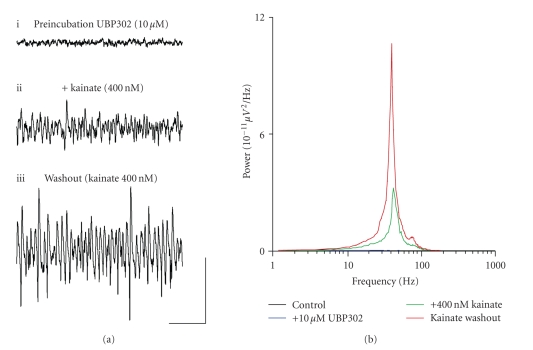
Preincubation of slices in UBP302 inhibits the ability of the MEC network to produce kainate-driven gamma frequency oscillations. (a) Extracellular field recordings showing 1 second epochs of activity (i) following preincubation with 10 *μ*M UBP302, (ii) following application of 400 nM kainate onto preincubated slices, and (iii) during a washout period into 400 nM kainate. (b) Pooled power spectra (*n* = 11) produced from 60 second epochs of extracellular field recorded data, showing a control recording (black), a recording following 10 *μ*M UBP302 preincubation (blue), 400 nM kainate application following preincubation (green), and washout into 400 nM kainate (red). Scale bar represents 200 milliseconds and 100 *μ*V.

First, we tested the ability of UBP302 to inhibit the generation of a kainate-driven gamma frequency oscillation in the MEC. Slices were preincubated in UBP302 (10 *μ*M) for 30 minutes. As expected, UBP302 administration caused no neuronal network activity in slices (*n* = 11) (Figure 3(a)). However, when kainate was applied to slices following preincubation with UBP302, gamma frequency oscillations were generated in all slices (*n* = 11) (Figure 3(a)). On washout into kainate alone (400 nM), although the frequency of oscillations did not change significantly (in presence of kainate following preincubation with UBP302, 45.4 ± 2.0 Hz; *v.* 400 nM kainate alone, 40.3 ± 0.9 Hz; *P* > .05; *n* = 11), oscillations were seen to increase significantly in both peak amplitude (in presence of kainate following preincubation with UBP302, 39.2 ± 12.1 *μ*V^2^/Hz; *v.* 400 nM kainate alone, 122.9 ± 32.8 *μ*V^2^/Hz; *P* < .05; *n* = 11) and area power (in presence of kainate following preincubation with UBP302, 545.1 ± 159.6 *μ*V^2^/Hz.Hz; *v.* 400 nM kainate alone, 1302.5 ± 241.4 *μ*V^2^/Hz.Hz; *P* < .05; *n* = 11) (Figures 3(a), 3(b)).

**Figure 4:**
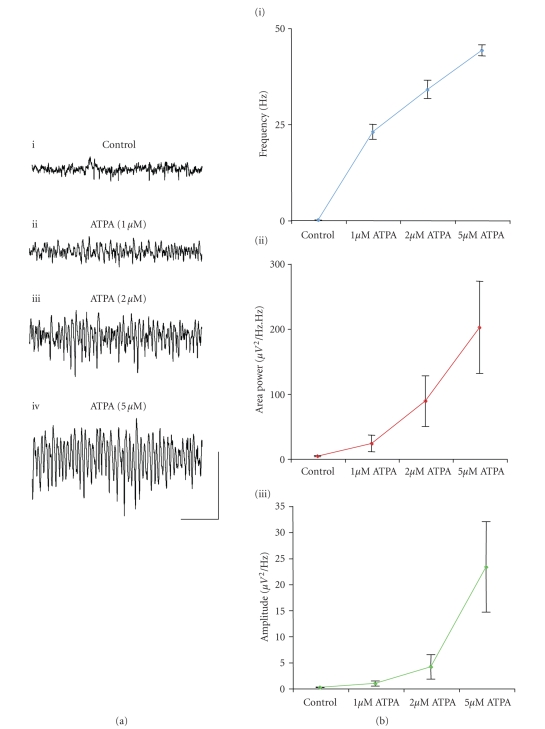
Activation of GLU_K5_-containing KARs can induce gamma frequency oscillations in the MEC. (a) Extracellular field recordings showing 1 second epochs of activity in a control setting (i) following application of 1 *μ*M, (ii) 2 *μ*M (iii), and 5 *μ*M ATPA (iv). (b) Pooled line graphs (*n* = 10) demonstrating the effects of varying ATPA concentration on (i) frequency, (ii) area power, and (iii) peak amplitude of gamma oscillations in the MEC. Scale bar represents 200 milliseconds and 100 *μ*V.

We next investigated whether gamma frequency oscillations could be generated in the MEC by application of the GLU_K5_ selective agonist, ATPA. ATPA was bath applied to slices at concentrations of 1 *μ*M, 2 *μ*M, and 5 *μ*M. ATPA induced gamma frequency oscillations in the MEC in the majority of slices to which the agonist was applied (*n* = 18 out of a total *n* = 26) (Figure 4(a)). Slices showing gamma oscillations upon ATPA application were observed to be dorsal MEC slices. Upon increasing the concentration of ATPA in slices demonstrating gamma oscillations, the mean frequency, peak amplitude, and area power of oscillations increased (*n* = 10) (Figures 4(a), 4(b)). The frequency of oscillations increased from 23.0 ± 1.9 Hz, to 34.0 ± 2.4 Hz, and to 44.2 ± 1.5 Hz (*n* = 10) (Figure 4(b)), the peak amplitude increased from 0.9 ± 0.5 *μ*V^2^/Hz, to 4.1 ± 2.3 *μ*V^2^/Hz, and to 23.3 ± 8.7 *μ*V^2^/Hz (*n* = 10) (Figure 4(b)), and the power increased from 23.1 ± 12.6 *μ*V^2^/Hz.Hz, to 88.4 ± 38.9 *μ*V^2^/Hz.Hz, and to 201.8 ± 71.0 *μ*V^2^/Hz.Hz, at the respective concentrations of ATPA (1 *μ*M, 2 *μ*M, and 5 *μ*M) (*n* = 10) (Figure 4(b)). Control readings, taken before ATPA administration, showed that no network activity was spontaneously present in slices (*n* = 26) (Figure 4(a)i). ATPA-induced gamma frequency oscillations were susceptible to the AMPA/KAR antagonist NBQX (10 *μ*M) (*n* = 3).

**Figure 5:**
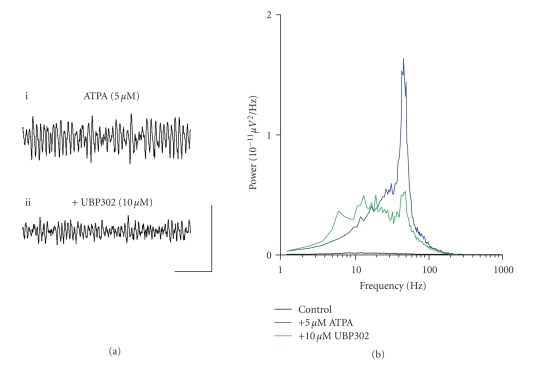
ATPA-generated gamma frequency oscillations in the MEC are reduced by application of the GLU_K5_ selective antagonist, UBP302. (a) Extracellular field recordings showing 1 second epochs of activity (i) in the presence of 5 *μ*M ATPA and (ii) following application of 10 *μ*M UBP302. (b) Pooled power spectra (*n* = 4) produced from 60 second epochs of extracellular field recorded data, showing a control recording (black), recording in the presence of 5 *μ*M ATPA (blue), and application of 10 *μ*M UBP302 (green). Scale bar represents 200 milliseconds and 100 *μ*V.

We next investigated the effect of UBP302 on ATPA-induced gamma frequency oscillations in the MEC. Gamma frequency oscillations were induced in slices by bath application of ATPA (2–5 *μ*M) (*n* = 4) (Figure 5(a)i). UBP302 (10 *μ*M) application caused no significant change in the frequency of gamma oscillations (control, 42.7 ± 3.9 Hz; *v.* UBP302, 33.9 ± 7.3 Hz; *P* > .1; *n* = 4) and yet had significant effects on both the peak amplitude (control, 20.8 ± 7.1 *μ*V^2^/Hz; *v.* UBP302, 6.6 ± 2.8 *μ*V^2^/Hz; *P* < .05; *n* = 4) and power of oscillations (control, 359.7 ± 117.9 *μ*V^2^/Hz.Hz; *v.* UBP302, 141.7 ± 61.9 *μ*V^2^/Hz.Hz; *P* < .05; *n* = 4) (Figures 5(a)ii, 5(b)). The effects of UBP302 on an ATPA-induced gamma frequency oscillations were not reversible on washout (*n* = 4).

### 3.4. A role for homomeric GLU_K5_-containing KARs in gamma frequency oscillations

The GLU_K5_ selective KAR antagonist, NS3763, was used to investigate the contribution of homomeric GLU_K5_-containing KARs to gamma activity in the MEC. NS3763 selectively antagonises homomeric GLU_K5_ KARs [39] and experiments were carried out to determine the role of these homomeric receptors in both kainate- and ATPA-induced gamma oscillations.

**Figure 6:**
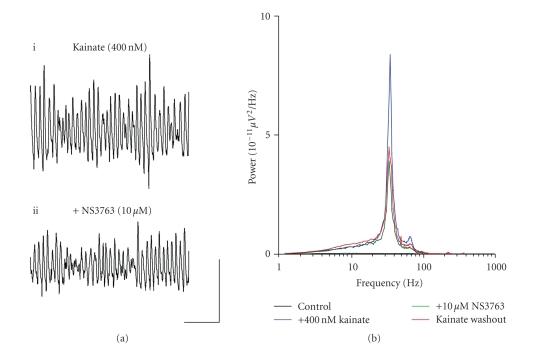
Blocking homomeric GLU_K5_-containing KARs significantly reduces kainate-driven gamma frequency oscillations in the MEC. (a) Extracellular field recordings showing 1 second epochs of activity (i) in the presence of 400 nM kainate and (ii) following application of 10 *μ*M NS3763. (b) Pooled power spectra (*n* = 8) produced from 60 second epochs of extracellular field recorded data, showing a control recording (black), recording in the presence of 400 nM kainate (blue), application of 10 *μ*M NS3763 (green), and a washout back into 400 nM kainate (red). Scale bar represents 200 milliseconds and 100 *μ*V.

Application of NS3763 (10–15 *μ*M) caused significant decreases in both peak amplitude (control, 100.2 ± 48.4 *μ*V^2^/Hz; *v.* NS3763, 46.0 ± 23.7 *μ*V^2^/Hz; *P* < .05; *n* = 8) and area power (control, 822.9 ± 273.2 *μ*V^2^/Hz.Hz; *v.* NS3763, 449.4 ± 182.3 *μ*V^2^/Hz.Hz; *P* < .05; *n* = 8) of kainate-induced gamma oscillations in the MEC (Figures 6(a), 6(b)). However, no effect was seen on the frequency of kainate-generated oscillations (control, 38.3 ± 2.7 Hz; *v.* NS3763, 36.0 ± 1.5 Hz; *P* > .1; *n* = 8) (Figure 6(b)).

**Figure 7:**
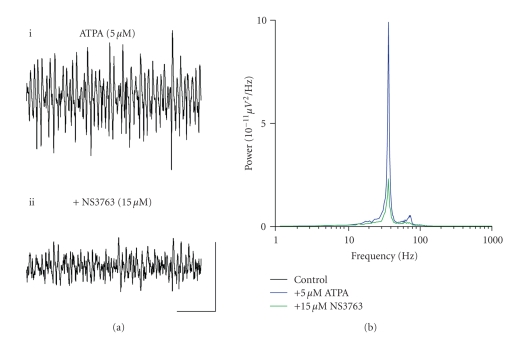
Blocking homomeric GLU_K5_-containing KARs effectively reduces power and amplitude of ATPA-generated gamma frequency oscillations in the MEC (a) Extracellular field recordings showing 1 second epochs of activity (i) in the presence of 5 *μ*M ATPA and (ii) following application of 15 *μ*M NS3763. (b) Pooled power spectra (*n* = 4) produced from 60 second epochs of extracellular field recorded data, showing a control recording (black), recording in the presence of 5 *μ*M ATPA (blue), and application of 15 *μ*M NS3763 (green). Scale bar represents 200 milliseconds and 100 *μ*V.

Application of NS3763 (10–15 *μ*M) to slices demonstrating ATPA-induced gamma oscillations caused no significant change in the frequency of oscillations (control, 46.7 ± 3.8 Hz; *v.* NS3763, 38.5 ± 6.2 Hz; *P* > .1; *n* = 8) (Figures 7(a), 7(b)). However, the presence of NS3763 caused a significant decrease in both the peak amplitude (control, 191.9 ± 63.1 *μ*V^2^/Hz; *v.* NS3763, 28.5 ± 13.6 *μ*V^2^/Hz; *P* < .05; *n* = 8) and area power (control, 1192.9 ± 342.8 *μ*V^2^/Hz.Hz; *v.* NS3763, 333.6 ± 120.5 *μ*V^2^/Hz.Hz; *P* < .05; *n* = 8) of gamma oscillations (Figures 7(a), 7(b)). The effects of NS3763 on either kainate- or ATPA-induced gamma frequency oscillations were not reversible on washout (*n* = 12).

### 3.5. A role for GLU_K5_-containing KARs in carbachol-induced gamma oscillations

**Figure 8:**
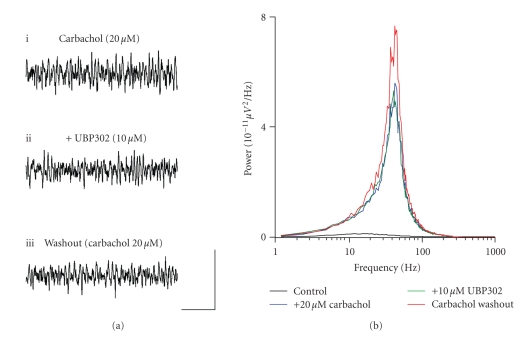
Carbachol-induced gamma frequency oscillations are not dependent on GLU_K5_-containing KARs. (a) Extracellular field recordings showing 1 second epochs of activity (i) in the presence of 20 *μ*M carbachol, (ii) following 10 *μ*M UBP302 application, and (iii) during a washout period into 20 *μ*M carbcahol. (b) Pooled power spectra (*n* = 6) produced from 60 second epochs of extracellular field recorded data, showing a control recording (black), recording in the presence of 20 *μ*M carbachol (blue), application of 10 *μ*M UBP302 (green), and washout back into 20 *μ*M carbachol (red). Scale bar represents 200 milliseconds and 100 *μ*V.

Cortical gamma frequency oscillations can also be induced by application of carbachol, an agonist at muscarinic acetylcholine receptors (mAChRs) [24, 25, 45–49]. It is unclear as to the role of GLU_K5_-containing KARs in a cholinergic-mediated gamma frequency oscillation in the MEC. Carbachol will cause an increase in the release of glutamate in the form of rhythmic EPSPs [46]. This, in turn, may lead to activation of KARs [50]. In agreement with previous studies in the MEC [24, 25] bath application of carbachol (10–20 *μ*M) generated persistent gamma frequency oscillations (*n* = 6) (Figure 8(a)i). Application of UBP302 (10 *μ*M) had no significant effect on the frequency (control, 41.7 ± 1.6 Hz; *v.* UBP302, 40.3 ± 0.6 Hz; *P* > .1; *n* = 6), peak amplitude (control, 5.9 ± 3.1 *μ*V^2^/Hz; *v.* UBP302, 5.5 ± 2.5 *μ*V^2^/Hz; *P* > .1; *n* = 6) or power (control, 155.2 ± 72.7 *μ*V^2^/Hz.Hz; *v.* UBP302, 148.7 ± 62.8 *μ*V^2^/Hz.Hz; *P* > .1; *n* = 6) of preestablished carbachol-driven gamma oscillations (Figures 8(a)ii, 8(b)). This lack of effect was further demonstrated by washout back into carbachol causing no significant change in observed gamma frequency oscillations (Figures 8(a)iii, 8(b)).

## 4. DISCUSSION

A number of studies have examined the contribution of various KAR subunits to gamma frequency oscillations in the hippocampus in vitro. Fisahn et al. [10] focused on the roles of GLU_K5_ and GLU_K6_ subunits in kainate-induced hippocampal gamma oscillations, using brain slices from transgenic GLU_K5_ and GLU_K6_ receptor knockout mice. Knockout of GLU_K5_ caused increased sensitivity of the hippocampal network to the effects of kainate and higher susceptibility to oscillatory and epileptogenic activity. Slices from GLU_K6_-knockout mice could not support kainate-induced gamma oscillations or epileptiform activity, suggesting distinct roles for GLU_K5_ and GLU_K6_ subunits in the hippocampus. Fisahn et al. [10] concluded that GLU_K5_-containing receptors may be expressed on axons of hippocampal interneurons and have a function in inhibitory tone, and that GLU_K6_-containing KARs may be found in the somatodendritic region of pyramidal cells and interneurons, and provide excitatory drive. Functional receptors of both subtypes must interact to allow generation of stable gamma oscillations in the hippocampus [9, 10].

Subsequently, Brown et al. [51] used pharmacological approaches to investigate the role of GLU_K5_-containing receptors in hippocampal gamma oscillations. This study used the GLU_K5_-selective agonists ATPA and iodowillardiine but found that neither could induce gamma network activity in area CA3 of rat hippocampal slices. The GLU_K5_ selective antagonist, UBP296, when preincubated with hippocampal slices, did not prevent induction of kainate-driven gamma oscillations. However, UBP296 produced an approximately 50% reduction in the power of preestablished kainate-induced gamma frequency oscillations. This paper concluded that GLU_K5_-containing KARs alone cannot generate gamma oscillations in the hippocampus but may be involved in maintenance of hippocampal gamma activity generated through other KAR subtypes.

In the present study, we have demonstrated that, similarly to in the hippocampus [51], GLU_K5_-containing KARs in the MEC have a role in the maintenance of kainate-driven oscillations. UBP302, a GLU_K5_ selective antagonist, caused reductions in peak amplitude and spectral power of preestablished kainate-induced gamma frequency oscillations in the MEC. Furthermore, pretreatment of slices with UBP302 partially inhibited generation of kainate-induced gamma frequency oscillations, suggesting that GLU_K5_-containing KARs are at least partially responsible for the induction of gamma oscillations by kainate application. These data suggest that in the MEC, differently to in the hippocampus [5, 8, 9], activation of GLU_K5_-containing KARs plays a role in the ability of MEC neuronal networks to generate gamma frequency oscillations. Moreover, in contrast to hippocampal gamma evoked by kainate, MEC gamma generated with GLU_K5_ agonists demonstrates a frequency increment with increased excitatory drive. This may reflect the manifestation of fundamentally different mechanisms of local circuit gamma oscillation generation in these two regions.

It was not surprising then that application of the GLU_K5_ subunit selective agonist ATPA [41] successfully evoked gamma frequency oscillations in the MEC. However, care must be taken with interpretation of this data as ATPA has recently been shown not to be entirely selective for GLU_K5_-containing KARs [40]. In fact, ATPA can activate both homomeric and heteromeric KAR complexes containing GLU_K5_, and also GLU_K6_/KA2 heteromeric KARs [48]. Thus, it cannot initially be assumed that these gamma oscillations have been generated via GLU_K5_-containing receptor complexes, since they could have been induced through GLU_K6_/KA2 heteromeric receptors. UBP302, however, is an antagonist with selectivity for GLU_K5_-containing KARs [36, 37]. Whilst UBP302 has been shown to block GLU_K7_ with an IC_50_ value of 4 *μ*M—this makes it ~10-fold selective for GLU_K5_ versus GLU_K7_—it does not have activity on GLU_K6_ or GLU_K6_/KA2 up to 100 *μ*M. Indeed, some controversy surrounds the activity of UBP302 on GLU_K7_ as it has been reported that UBP302 failed to demonstrate any potent activity in a binding assay on GLU_K7_ (personal communication, D.E. Jane). In any case, as UBP302 only blocks homomeric GLU_K7_ and activation of GLU_K7_ requires very high glutamate concentrations (EC_50_ value 5.9 mM) [52] it may not be relevant to this study. Application of UBP302 onto slices showing ATPA-generated network activity causes reduced peak amplitude and an approximately 60% reduction in area power of gamma frequency oscillations. This inhibition of ATPA-generated gamma oscillations by UBP302 suggests that the observed activity must, at least in part, be due to activation of GLU_K5_-containing KARs.

Moreover, NS3763 application caused a significant reduction in peak amplitude and spectral power of preestablished kainate-driven gamma oscillations. This demonstrates that homomeric GLU_K5_-containing KARs are at least partially responsible for the maintenance of these kainate-driven gamma frequency oscillations. Application of NS3763 to preestablished ATPA-generated oscillations caused an approximately 80% reduction in area power of gamma frequency oscillations and also a reduction in peak amplitude. This suggests that a large component of an ATPA-driven gamma oscillation is maintained through homomeric GLU_K5_ KARs. The activity of the selective homomeric GLU_K5_-containing KAR antagonist, NS3763, on both kainate- and ATPA-generated gamma frequency oscillations, tells us that homomeric GLU_K5_-containing KARs are involved in the observed network activity.

It has been suggested that carbachol-driven activity could cause excess glutamate release and that this overspill of glutamate could activate KARs [50]. The lack of effect of the GLU_K5_ selective antagonist, UBP302, on carbachol-induced gamma oscillations in the MEC suggests that GLU_K5_-containing KARs are not involved in the generation or maintenance of gamma oscillations induced via activation of muscarinic cholinergic receptor. However, we cannot rule out the possibility that other KAR subtypes may be involved in these mAChR-mediated gamma oscillations.

We have shown that GLU_K5_-containing KARs are implicated in the generation and maintenance of gamma frequency oscillations in the MEC evoked by kainate. However, we can only speculate on the cellular localisation of these GLU_K5_-containing receptors in the MEC. Research performed by Christensen et al. [40] in the hippocampus, suggested likely localisations of KAR subtypes in hippocampal CA1 inhibitory interneurons terminating with pyramidal cells, concluding that heteromeric GLU_K6_/KA2 receptors are expressed in somatodendritic compartments of interneurons, and that GLU_K5_ complexes, with either GLU_K6_ or KA2, are found at presynaptic terminals. It seems likely from our results that in the MEC, both homomeric and heteromeric GLU_K5_-containing KARs are present.

Presynaptic KARs are involved in regulation and modulation of neurotransmitter release at inhibitory and excitatory synapses in the hippocampus [50, 53, 54]. In contrast, postsynaptic KARs mediate excitatory postsynaptic currents (EPSCs) in many brain regions [35, 55]. KAR activation in the hippocampus modulates GABA release at terminals of inhibitory interneurons and causes an increase in spontaneous IPSCs but a reduction in the amplitude of these IPSCs impinging on to CA1 interneurons [40]. This suggests that KARs may be present in two distinct populations in hippocampal inhibitory interneurons, and the same may be true of KARs in the MEC [2, 40]. However, other reports have demonstrated that kainate can increase the frequency and amplitude of spontaneous IPSCs, but not action potential-independent miniature IPSCs in stratum radiatum interneurons [56]. Moreover, these authors also observed that kainate can directly depolarise the axonal plexus of inhibitory interneurons producing both increased antidromic and presumably orthodromic spiking. This effect would explain the ability of KAR activation to increase spontaneous but not miniature IPSC activity. The presence of KARs at an axonal loci has been well documented in the hippocampus, most notably in mossy fibres [57, 58].

As outlined in the previous paragraph, there is a large corpus of data on the role of KAR in the hippocampus. However, with respect to the MEC there is a paucity of such information. In order to put the current results presented in this paper into context, future work will concentrate on combining intracellular recordings from individual neurones (pyramidal and interneuron), specific pharmacological KAR tools, and transgenic KAR subunit knockout animals [9, 10] to elucidate the exact nature of cellular expression of KARs in the MEC.

## References

[1] Hollmann M, Heinemann S (1994). Cloned glutamate receptors. *Annual Review of Neuroscience*.

[2] Lerma J, Paternain AV, Rodríguez-Moreno A, López-García JC (2001). Molecular physiology of kainate receptors. *Physiological Reviews*.

[3] Wisden W, Seeburg PH (1993). A complex mosaic of high-affinity kainate receptors in rat brain. *The Journal of Neuroscience*.

[4] Bettler B, Mulle C (1995). Neurotransmitter receptors II. AMPA and kainate receptors. *Neuropharmacology*.

[5] Werner P, Voigt M, Keinanen K, Wisden W, Seeburg PH (1991). Cloning of a putative high-affinity kainate receptor expressed predominantly in hippocampal CA3 cells. *Nature*.

[6] Herb A, Burnashev N, Werner P, Sakmann B, Wisden W, Seeburg PH (1992). The KA-2 subunit of excitatory amino acid receptors shows widespread expression in brain and forms ion channels with distantly related subunits. *Neuron*.

[7] Chittajallu R, Braithwaite SP, Clarke VRJ, Henley JM (1999). Kainate receptors: subunits, synaptic localization and function. *Trends in Pharmacological Sciences*.

[8] Cui C, Mayer ML (1999). Heteromeric kainate receptors formed by the coassembly of GluR5, GluR6, and GluR7. *The Journal of Neuroscience*.

[9] Fisahn A (2005). Kainate receptors and rhythmic activity in neuronal networks: hippocampal gamma oscillations as a tool. *The Journal of Physiology*.

[10] Fisahn A, Contractor A, Traub RD, Buhl EH, Heinemann SF, McBain CJ (2004). Distinct roles for the kainate receptor subunits GluR5 and GluR6 in kainate-induced hippocampal gamma oscillations. *The Journal of Neuroscience*.

[11] Bragin A, Jandó G, Nádasdy Z, Hetke J, Wise K, Buzsáki G (1995). Gamma (40–100 Hz) oscillation in the hippocampus of the behaving rat. *The Journal of Neuroscience*.

[12] Chrobak JJ, Buzsáki G (1998). Gamma oscillations in the entorhinal cortex of the freely behaving rat. *The Journal of Neuroscience*.

[13] Csicsvari J, Jamieson B, Wise KD, Buzsáki G (2003). Mechanisms of gamma oscillations in the hippocampus of the behaving rat. *Neuron*.

[14] Engel AK, Singer W (2001). Temporal binding and the neural correlates of sensory awareness. *Trends in Cognitive Sciences*.

[15] Singer W (1993). Synchronization of cortical activity and its putative role in information processing and learning. *Annual Review of Physiology*.

[16] Gray CM, König P, Engel AK, Singer W (1989). Oscillatory responses in cat visual cortex exhibit inter-columnar synchronization which reflects global stimulus properties. *Nature*.

[17] Fell J, Klaver P, Lehnertz K (2001). Human memory formation is accompanied by rhinal-hippocampal coupling and decoupling. *Nature Neuroscience*.

[18] Fell J, Klaver P, Elger CE, Fernández G (2002). The interaction of rhinal cortex and hippocampus in human declarative memory formation. *Reviews in the Neurosciences*.

[19] Muller D, Nikonenko I, Jourdain P, Alberi S (2002). LTP, memory and structural plasticity. *Current Molecular Medicine*.

[20] Singer W, Gray CM (1995). Visual feature integration and the temporal correlation hypothesis. *Annual Review of Neuroscience*.

[21] Traub RD, Spruston N, Soltesz I, Konnerth A, Whittington MA, Jefferys JGR (1998). Gamma-frequency oscillations: a neuronal population phenomenon, regulated by synaptic and intrinsic cellular processes, and inducing synaptic plasticity. *Progress in Neurobiology*.

[22] Hirai N, Uchida S, Maehara T, Okubo Y, Shimizu H (1999). Enhanced gamma (30–150 Hz) frequency in the human medial temporal lobe. *Neuroscience*.

[23] Uchida S, Maehara T, Hirai N, Okubo Y, Shimizu H (2001). Cortical oscillations in human medial temporal lobe during wakefulness and all-night sleep. *Brain Research*.

[24] Van der Linden S, Panzica F, de Curtis M (1999). Carbachol induces fast oscillations in the medial but not in the lateral entorhinal cortex of the isolated guinea pig brain. *Journal of Neurophysiology*.

[25] Dickson CT, Biella G, de Curtis M (2000). Evidence for spatial modules mediated by temporal synchronization of carbachol-induced gamma rhythm in medial entorhinal cortex. *The Journal of Neuroscience*.

[26] Cunningham MO, Davies CH, Buhl EH, Kopell N, Whittington MA (2003). Gamma oscillations induced by kainate receptor activation in the entorhinal cortex in vitro. *The Journal of Neuroscience*.

[27] Cunningham MO, Halliday DM, Davies CH, Traub RD, Buhl EH, Whittington MA (2004). Coexistence of gamma and high-frequency oscillations in rat medial entorhinal cortex in vitro. *The Journal of Physiology*.

[28] Cunningham MO, Hunt J, Middleton S (2006). Region-specific reduction in entorhinal gamma oscillations and parvalbumin-immunoreactive neurons in animal models of psychiatric illness. *The Journal of Neuroscience*.

[29] Whittington MA, Traub RD, Jefferys JGR (1995). Synchronized oscillation in interneuron networks driven by metabotropic glutamate receptor activation. *Nature*.

[30] Hájos N, Pálhalini J, Mann EO, Nèmeth B, Paulsen O, Freund TF (2004). Spike timing of distinct types of GABAergic interneuron during hippocampal gamma oscillations in vitro. *The Journal of Neuroscience*.

[31] Gloveli T, Dugladze T, Saha S (2005). Differential involvement of oriens/pyramidale interneurones in hippocampal network oscillations in vitro. *The Journal of Physiology*.

[32] Whittington MA, Traub RD (2003). *Interneuron Diversity series*: inhibitory interneurons and network oscillations in vitro. *Trends in Neurosciences*.

[33] Cunningham MO, Whittington MA, Bibbig A (2004). A role for fast rhythmic bursting neurons in cortical gamma oscillations in vitro. *Proceedings of the National Academy of Sciences of the United States of America*.

[34] Bureau I, Bischoff S, Heinemann SF, Mulle C (1999). Kainate receptor-mediated responses in the CA1 field of wild-type and GluR6-deficient mice. *The Journal of Neuroscience*.

[35] Cossart R, Esclapez M, Hirsch JC, Bernard C, Ben-Ari Y (1998). GluR5 kainate receptor activation in interneurons increases tonic inhibition of pyramidal cells. *Nature Neuroscience*.

[36] More JCA, Nistico R, Dolman NP (2004). Characterisation of UBP296: a novel, potent and selective kainate receptor antagonist. *Neuropharmacology*.

[37] Dolman NP, Troop HM, More JCA (2005). Synthesis and pharmacology of willardiine derivatives acting as antagonists of kainate receptors. *Journal of Medicinal Chemistry*.

[38] Perrais D, Pineiro PS, Jane DE, Mulle C Antagonism of recombinant and native GluR7-containing receptors: new tools to study presynaptic kainate receptors.

[39] Christensen JK, Varming T, Ahring PK, Jorgensen TD, Nielsen EO (2004). In vitro characterisation of 5-carbozyl-2,4-di-benzamido-benzoic acid (NS3763), a non-competitive antagonist of GLU_K5_ receptors. *The Journal of Pharmacology and Experimental Theraputics*.

[40] Christensen JK, Paternain AV, Selak S, Ahring PK, Lerma J (2004). A mosaic of functional kainate receptors in hippocampal interneurons. *The Journal of Neuroscience*.

[41] Clarke VRJ, Ballyk BA, Hoo KH (1997). A hippocampal GluR5 kainate receptor regulating inhibitory synaptic transmission. *Nature*.

[42] Clarke VRJ, Collingridge GL (2002). Characterisation of the effects of ATPA, a GLU_K5_ receptor selective agonist, on excitatory synaptic transmission in area CA1 of rat hippocampal slices. *Neuropharmacology*.

[43] Clarke VRJ, Collingridge GL (2004). Characterisation of the effects of ATPA, a GLU_K5_ kainate receptor agonist, on GABAergic synaptic transmission in the CA1 region of rat hippocampal slices. *Neuropharmacology*.

[44] Campbell SL, Mathew SS, Hablitz JJ (2007). Pre- and postsynaptic effects of kainate on layer II/III pyramidal cells in rat neocortex. *Neuropharmacology*.

[45] Fisahn A, Pike FG, Buhl EH, Paulsen O (1998). Cholinergic induction of network oscillations at 40 Hz in the hippocampus in vitro. *Nature*.

[46] Traub RD, Bibbig A, Fisahn A, Lebeau FEN, Whittington MA, Buhl EH (2000). A model of gamma-frequency network oscillations induced in the rat CA3 region by carbachol in vitro. *European Journal of Neuroscience*.

[47] Hormuzdi SG, Pais I, LeBeau FEN (2001). Impaired electrical signaling disrupts gamma frequency oscillations in connexin 36-deficient mice. *Neuron*.

[48] Pálhalmi J, Paulsen O, Freund TF, Hájos N (2004). Distinct properties of carbachol- and DHPG-induced network oscillations in hippocampal slices. *Neuropharmacology*.

[49] Mann EO, Suckling JM, Hajos N, Greenfield SA, Paulsen O (2005). Perisomatic feedback inhibition underlies cholinergically induced fast network oscillations in the rat hippocampus in vitro. *Neuron*.

[50] Lauri SE, Segerstråle M, Vesikansa A (2005). Endogenous activation of kainate receptors regulates glutamate release and network activity in the developing hippocampus. *The Journal of Neuroscience*.

[51] Brown JT, Teriakidis A, Randall AD (2006). A pharmacological investigation of the role of GLU_K5_-containing receptors in kainate-driven hippocampal gamma band oscillations. *Neuropharmacology*.

[52] Schiffer HH, Swanson GT, Heinemann SF (1997). Rat GluR7 and a carboxy-terminal splice variant, GluR7b, are functional kainate receptor subunits with a low sensitivity to glutamate. *Neuron*.

[53] Contractor A, Swanson G, Heinemann SF (2001). Kainate receptors are involved in short- and long-term plasticity at mossy fiber synapses in the hippocampus. *Neuron*.

[54] Contractor A, Sailer AW, Darstein M (2003). Loss of kainate receptor-mediated heterosynaptic facilitation of mossy-fiber 
synapses in KA2^−/−^ mice. *The Journal of Neuroscience*.

[55] Vignes M, Collingridge GL (1997). The synaptic activation of kainate receptors. *Nature*.

[56] Semyanov A, Kullmann DM (2001). Kainate receptor-dependent axonal depolarization and action potential initiation in interneurons. *Nature Neuroscience*.

[57] Kamiya H, Ozawa S (2000). Kainate receptor-mediated presynaptic inhibition at the mouse hippocampal mossy fibre synapse. *The Journal of Physiology*.

[58] Schmitz D, Frerking M, Nicoll RA (2000). Synaptic activation of presynaptic kainate receptors on hippocampal mossy fiber synapses. *Neuron*.

